# Severe neonatal hypotonia due to *SLC30A5* variant affecting function of ZnT5 zinc transporter

**DOI:** 10.1002/jmd2.12465

**Published:** 2025-01-09

**Authors:** Vadim Dolgin, Pauline Chabosseau, Jacob Bistritzer, Iris Noyman, Orna Staretz‐Chacham, Ohad Wormser, Noam Hadar, Marina Eskin‐Schwartz, Bibi Kanengisser‐Pines, Ginat Narkis, Ramy Abramsky, Eilon Shany, Guy A. Rutter, Kyla Marks, Ohad S. Birk

**Affiliations:** ^1^ The Morris Kahn Laboratory of Human Genetics, Faculty of Health Sciences Ben Gurion University Beer‐Sheva Israel; ^2^ CRCHUM and Department of Medicine Université de Montréal Montréal QC Canada; ^3^ Pediatric Neurology Unit, Division of Pediatrics, Soroka University Medical Center, Faculty of Health Sciences Ben‐Gurion University of the Negev Beer Sheva Israel; ^4^ Department of Neonatology, Soroka University Medical Center, Faculty of Health Sciences Ben‐Gurion University of the Negev Beer Sheva Israel; ^5^ Metabolic Clinic, Division of Pediatrics, Soroka University Medical Center, Faculty of Health Sciences Ben‐Gurion University of the Negev Beer Sheva Israel; ^6^ Soroka Medical Center Genetics Institute Beer‐Sheva Israel; ^7^ Department of Diabetes, Endocrinology and Medicine, Faculty of Medicine Imperial College London UK; ^8^ LKC School of Medicine Nanyang Technological College Singapore Singapore; ^9^ The Danek Gertner Institute of Human Genetics Sheba Medical Center Tel‐Hashomer Ramat Gan Israel

**Keywords:** hypotonia, mutation, neurological syndrome, SLC30A5, ZnT5

## Abstract

The tightly‐regulated spatial and temporal distribution of zinc ion concentrations within cellular compartments is controlled by two groups of Zn^2+^ transporters: the 14‐member ZIP/SLC39 family, facilitating Zn^2+^ influx into the cytoplasm from the extracellular space or intracellular organelles; and the 10‐member ZnT/SLC30 family, mobilizing Zn^2+^ in the opposite direction. Genetic aberrations in most zinc transporters cause human syndromes. Notably, previous studies demonstrated osteopenia and male‐specific cardiac death in mice lacking the ZnT5/*SLC30A5* zinc transporter, and suggested association of two homozygous frameshift *SLC30A5* variants with perinatal mortality in humans, due to hydrops fetalis and hypertrophic cardiomyopathy. We set out to decipher the molecular basis of a severe hypotonia syndrome. Combining homozygosity mapping and exome sequencing studies of consanguineous Bedouin kindred, as well as transfection experiments and zinc monitoring in HEK293 cells, we demonstrate that a bi‐allelic in‐frame 3bp deletion variant in *SLC30A5*, deleting isoleucine within the highly conserved cation efflux domain of the encoded ZnT5, results in lower cytosolic zinc concentrations, causing a syndrome of severe non‐progressive neonatal axial and limb hypotonia with high‐arched palate and respiratory failure. There was no evidence of hydrops fetalis, cardiomyopathy or multi‐organ involvement. Affected infants required nasogastric tube or gastrostomy feeding, suffered from various degrees of respiratory compromise and failure to thrive and died in infancy. Thus, a biallelic variant in *SLC30A5* (ZnT5), affecting cytosolic zinc concentrations, causes a severe hypotonia syndrome with respiratory insufficiency and failure to thrive, lethal by 1 year of age.

**Synopsis**

Biallelic SLC30A% / ZNT variant affecting zinc transport causes a syndrome of severe non‐progressive neonatal axial and limb hypotonia with high‐arched palate and respiratory failure.

## 1 INTRODUCTION

Zinc, a redox‐inert trace element that is second only to iron in abundance in all living organisms, is confined within cellular compartments to avoid cytotoxicity,^1^ and serves as a cofactor in over 300 enzymes spanning all enzyme classes.^2^ Zinc concentrations are highest in the hippocampus and neocortex of the brain, prostate gland, and islets of Langerhans of the pancreas, with uneven distributions.^1^ The spatio‐temporal intracellular dynamics of zinc, crucial in cellular signaling, are mediated through two prominent groups of Zn^2+^ transporters: the 14‐member Zrt, Irt‐like Protein (ZIP) family, encoded by *SLC39A1–14*, facilitates Zn^2+^ influx into the cytoplasm from the extracellular space or intracellular organelles^3^; and the Zinc Transporter (ZnT) family, with its 10 members encoded by *SLC30A1–10*, mobilizes Zn^2+^ in the opposite direction, removing excess zinc from the cytoplasm.^4^ The various mammalian zinc transporters are found in specific subcellular locations, enriching zinc in the lumen of specific subcellular compartments to support zinc‐dependent cellular processes, safeguard cellular homeostasis, and modulate cellular responsiveness to pathophysiologic signals.^1^

Mutations in several zinc transporters have been shown to cause different pathological conditions and syndromes^5^, ^6^, ^7^, ^8^, ^9^, ^10^, ^11^: *ZIP2* polymorphism is associated with susceptibility to carotid artery disease; biallelic *ZIP4* mutations cause acrodermatitis enterohepatica; *ZIP7* mutations cause autosomal recessive agammaglobulinemia; *ZIP8* biallelic mutations cause congenital disorder of glycosylation type IIn; and *ZIP13* mutations cause an autosomal recessive spondylo‐cheirodysplastic form of Ehlers Danlos syndrome. Human diseases have been shown to be caused also by variants in ZnT proteins: heterozygous *ZnT2* mutations cause transient neonatal zinc deficiency; *ZnT8* polymorphism has been associated with augmented risk of type 2 diabetes; and, as we have previously shown,^12^ biallelic *ZnT9* (*SLC30A9*) mutation causes Birk‐Landau‐Perez cerebro‐renal syndrome (OMIM 604604), characterized by global developmental delay from infancy or early childhood. Notably, autosomal recessive *ZnT10* biallelic mutations have been shown to cause hypermanganesemia, Parkinsonism and dystonia, by disturbing homeostasis of manganese, rather than of zinc.^13^ We now demonstrate through genetic and functional studies, that a syndrome of severe neonatal hypotonia and respiratory compromise, culminating in death by 1 year of age, can be caused by biallelic variant in ZnT5 (encoded by *SLC30A5*), resulting in significantly diminished cytosolic free zinc levels.

## 2 MATERIALS AND METHODS

### 2.1 Ethics statement

The study was approved by Soroka Medical Center's institutional review board (approval #5071G) and the Israel Ministry of Health National Helsinki committee (approval #920100319). All procedures were in accordance with the 1964 Helsinki declaration and its later amendments or comparable ethical standards. Written informed consent was obtained from all individuals studied or their legal guardians.

### 2.2 Clinical phenotyping

Affected individuals were examined by an experienced team of geneticists, pediatricians, and neurologists. Clinical assessment was also done for non‐affected parents and siblings as needed.

### 2.3 Genomic DNA extraction from whole blood

Blood samples were obtained from patients and healthy family members. Blood samples (3–10 mL) were collected in BD™ EDTA tubes, and total genomic DNA was extracted from leukocytes of peripheral blood samples using E.Z.N.A.® Blood DNA Kit salting out procedure and isopropanol precipitation. DNA concentrations were determined by spectrophotometer at OD260 and ran on 1% agarose gel in TBE to validate its integrity (expected to be ~20 kb in size).

### 2.4 Genome‐wide linkage analysis

Genome‐wide linkage analysis was performed using Single Nucleotide Polymorphism (SNP) microarrays as previously described. In some detail, DNA samples of the three affected individuals, their parents, and healthy sibling of each of the two families studied were assayed using the Illumina® Infinium® OmniExpress 24BeadChip Array, testing approximately 740 000 SNPs across the genome. Homozygosity by descent analysis was carried out using HomozygosityMapper,^14^ assuming an autosomal recessive mode of inheritance of the phenotype and a founder mutation. All physical positions mentioned are according to the GRCh37/hg19 genome assembly.

### 2.5 Whole‐exome sequencing

Whole exome sequencing (WES) was performed for four affected individuals and parents of the affected infant of the third family (trio) as previously described.^15^, ^16^ Briefly, WES was done using paired‐end (2 × 101) protocol at a mean coverage of 30‐fold (HiSeq2000, Illumina, Theragene Etex, Republic of Korea). Of all exonic nucleotides, 85%–90% were covered by >10 reads. For exome enrichment, we used SureSelect XT V5 Library Prep Kit targeting 51 Mb regions. Sequencing, read alignment, variant calling, and annotation were performed by Theragen Etex Bioinformatics Team. WES data were then analyzed using QIAGEN's Ingenuity® Variant Analysis™ software (http://www.qiagenbioinformatics.com/ingenuity-variant-analysis; QIAGEN, Redwood City), excluding variants observed with an allele frequency ≥1% in the 1000 Genomes Project (http://www.internationalgenome.org/) or the Allele Frequency Community (http://www.allelefrequencycommunity.org/), or variants appearing in a homozygous state in our in‐house WES database of 500 ethnically matched controls. Variants were then further sifted based on their being predicted deleterious (as listed in HGMD® or ClinVar), previously classified as disease‐associated (pathogenic or likely pathogenic) according to computed ACMG guidelines classification, or being associated with loss of function by causing frameshift, in‐frame indel, start/stop codon change, missense or splice site loss up to five bases into an intron (as predicted by MaxEntScan). Of the remaining variants, we selected only those within the chromosome five disease‐associated locus and segregating within the family as expected for autosomal recessive heredity (assayed through Sanger sequencing).

### 2.6 Multiple sequence alignment

ClustalW2 is a multiple sequence alignment computer program hosted at the European Bioinformatics Institute (EMBL‐EBI) website (http://www.ebi.ac.uk/Tools/msa/clustalw2/).^17^ Protein alignments used: *H. sapiens*—NP_075053.2; *P. troglodytes*—XP_001161060.2; *M. mulatta*—XP_001090696.1; *C. lupus*—XP_850350.1; *B. taurus*—NP_001179103.1; *M. musculus*—NP_075023.2; *R. norvegicus*—NP_001099874.1; *G. gallus*—NP_001026590.2; *D. rerio*—NP_001002322.1; *X. tropicalis*—NP_001015911.1; *C. elegans*—NP_740931.2.

### 2.7 Verification of variants identified and population screening

First, the Integrative Genomics Viewer (IGV)^18^, ^19^ was used to directly visualize genomic loci of particular interest and the variants arising from the WES. Next, the disease‐associated variant identified was assayed in all affected and unaffected family members using Sanger sequencing. Sanger sequencing was performed using ABI PRISM 3730 DNA Analyzer (Applied Biosystems, USA) with specific primers designed using Primer‐Blast^20^: *SLC30A5*‐F: 5′‐gcagcattggtgtgatcgtat‐3′ and *SLC30A5*‐R: 5′‐catattctggtggcaatctcagga‐3′.

### 2.8 Cloning constructs

The full *SLC30A5* (NM_022902.5) transcript (2298 bp) was PCR‐amplified from cDNA of a healthy individual using a set of primers, creating overlapping ends with a cloning site of a vector described below: SLC30A5_ins_F 5′‐TGCAGAATTCGCCACCatggaggagaaatacg‐3′ and SLC30A5_ins_R 5′‐CGAGCTCGGATCCtcacatgatgtaagtaccatctttgcagtatttca‐3′. Using the Gibson assembly protocol, this insert was ligated into the PCR‐linearized pCDNA™ 3.1 (−) expression vector (Invitrogen). Primers for linearization PCR were: SLC30A5_vector_F 5′‐tacatcatgtgaGGATCCGAGCTCGGTACCA‐3′ and SLC30A5_vector_R 5′‐tccatGGTGGCGAATTCTGCAGATATCCAGCACAGTGG‐3′. The c.1897_1899delATA variant was introduced by DpnI‐mediated site‐directed mutagenesis (PCR with primers SLC30A5_mut_F 5′‐tttctcagtgttgttccac‐3′ and SLC30A5_mut_R 5′‐taatatagcaataaaaagagaacag‐3′ followed by ligation). Both wild type (WT) and mutant (Mut) plasmids were purified and verified by Sanger sequencing.

### 2.9 Zinc measurements

Zinc measurements were performed as previously described.^21^ Briefly, HEK293 cells were grown in Dulbecco's modified Eagle medium containing 10% foetal calf serum, 2 mM L‐glutamine, 100 units/mL penicillin, and 100 mg/mL streptomycin at 37°C in a humidified atmosphere containing 5% CO_2_. Cells were plated on sterile coverslips in 6‐well plates at 50%–60% confluence and co‐transfected with 1.0 μg of eCALWY4‐pShuttle plasmid DNA and with 1.0 μg of plasmid for ZnT5‐WT or ZnT5‐mut expression by using Lipofectamine 2000 (Invitrogen) following the manufacturers' instructions. Cells were then allowed to express proteins for 16–24 h. Cells were then washed in Krebs‐HEPES‐bicarbonate (KHB) buffer (140 mM NaCl, 3.6 mM KCl, 0.5 mM NaH_2_PO_4_, 0.2 mM MgSO_4_, 1.5 mM CaCl_2_, 10 mM HEPES, 25 mM NaHCO_3_), which was warmed, bubbled with 95:5 O_2_/CO_2_, set to pH 7.4, and contained 11 mM glucose. Cells were maintained at 37°C throughout with a heating stage (MC60, LINKAM, Scientific Instruments), and KHB was perfused (1–1.5 mL/min) with additions as stated in Figure 3. Images were captured at 433 nm monochromatic excitation wavelength (Polychrome IV, Till photonics) using an Olympus IX‐70 wide‐field microscope with a 40×/1.35 NA oil immersion objective and a Zyla sCMOS camera (Andor Technology) controlled by Micromanager software. Emitted light was split and filtered by a Dual‐View beam splitter (Photometrics) equipped with a 505dcxn dichroic mirror and two emission filters (Chroma Technology, D470/24 for cerulean and D535/30 for citrine). Image analysis was performed with ImageJ software using a homemade macro, and the fluorescence emission ratios were derived after subtracting the background. Steady‐state fluorescence intensity ratio citrine/cerulean (*R*) was measured, then maximum and minimum ratios were determined to calculate free Zn^2+^ concentration using the following formula: [Zn^2+^] = *K*_d_(*R*_max_ − *R*)/(*R* − *R*_min_). The maximum ratio (*R*_max_) was obtained upon intracellular zinc chelation with 50 mM TPEN, and the minimum ratio (*R*_min_) was obtained upon zinc saturation with 100 mM ZnCl_2_ in the presence of the Zn^2+^ ionophore, pyrithione (5 mM). For these experiments, we used the eCALWY‐4 member of the eCALWYs probe family, which *K*_d_ (630 pM) is the most accurate for in cellulo zinc measurement.

## 3 RESULTS

### 3.1 Delineation of the disease phenotype

**FIGURE 1:**
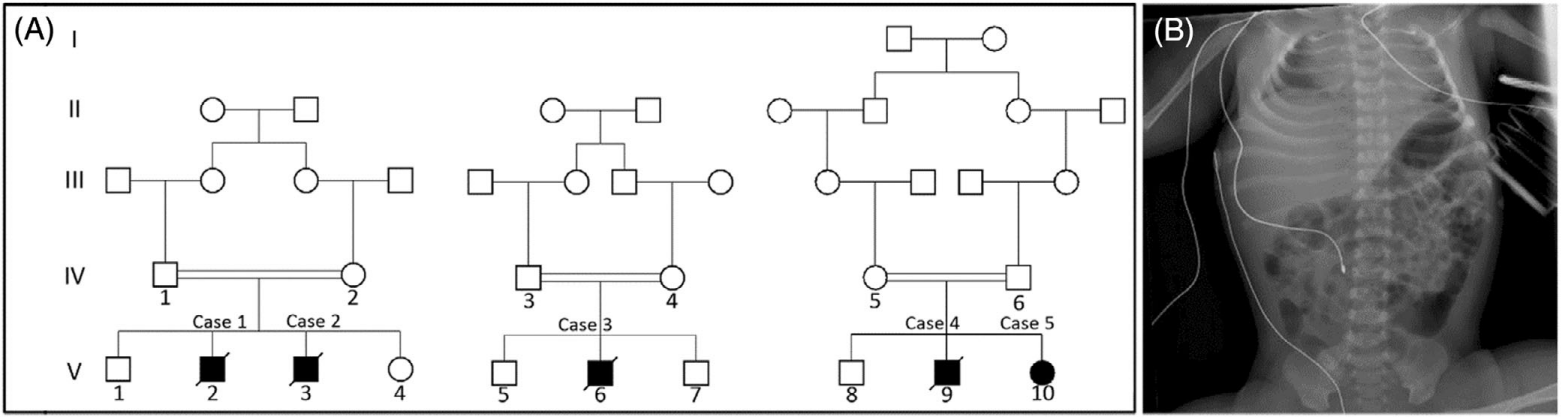
The disease phenotype: (A) family tree of the affected kindred. (B) Chest X‐ray of affected individual V‐3, showing restricted lung expansion (less than five ribs) and upwardly bilaterally displaced diaphragm.

Five infants (four male and one female) of three closely‐related consanguineous families of the same inbred Bedouin tribe, presented at birth with an apparently autosomal recessive disease (Figure 1A). All five infants were born following normal pregnancies, without polyhydramnios or reduced fetal movements, at gestational ages ranging from late preterm (35^+6^ weeks) to post‐term (42^+6^ weeks). Birth weights (2.445–3.965 kg) were appropriate for gestational age. All five presented at birth with severe axial and limb non‐progressive hypotonia with a high arched palate, severe head lag, poor suck, and weak neonatal primitive reflexes, as well as varying degrees of respiratory effort and feeding difficulties. Head circumference was normal with a large anterior fontanelle in most. Deep tendon reflexes were normal or brisk and there were no tongue fasciculations, muscle wasting, seizures, cardiac or multiorgan involvement. Two of the infants (V‐6, V‐10) required intubation and mechanical ventilation within 24 h of birth following hypoxia and respiratory acidosis. Both demonstrated small lung volumes; less than five ribs and upwardly displaced diaphragms bilaterally on chest X‐rays (Figure 1B). One of those two infants (V‐10), born after prolonged ruptured membranes, suffered from severe pulmonary hypertension and required nitric oxide treatment; however, within 2 days, both ventilated infants had normal blood gases and did not require supplemental oxygen, confirming the absence of significant pulmonary hypoplasia. Both ventilated infants failed repeated extubation attempts using different non‐invasive ventilation modes and eventually underwent tracheostomy for prolonged ventilation. One of the ventilated infants (V‐6) succumbed due to massive aspiration pneumonia, cardiac arrest, and severe anoxic brain injury at age 1 year. All affected individuals required nasogastric tube or gastrostomy feeds, and all had failure to thrive (FTT). None of the affected individuals survived beyond 1 year, and only one infant (V‐10), age 9 months, is still alive. Although detailed standard neurodevelopmental assessment was difficult to perform given the severe hypotonia and failure to thrive, all infants had moderate to severe developmental delay. Extensive laboratory investigations, including urinary organic acids, blood biochemistry (including amino acids, cholesterol, transferrin iso‐electric focusing, very long chain fatty acids, lactate/pyruvate, carnitine, magnesium, blood gases, creatinine phosphokinase, and glucose), endocrine tests (including growth hormone, TSH, prolactin), and genetic screening for known muscular dystrophies, myopathies, and myasthenic syndromes, specific testing for Prader Willi, spinal muscular atrophy (SMA), and myotonic dystrophy, as well as karyotype and chromosomal microarrays (CMA)—ruled out known relevant diseases.

Four of the affected infants had brain MRIs, with non‐consistent nonspecific findings, such as varying degrees of thinning of the corpus callosum, increased extra‐axial space, mega‐cisterna magna, and mild ventriculomegaly, possibly suggestive of cerebral atrophy. Muscle biopsy, performed for V‐6, demonstrated non‐specific mild myopathic changes and, on electron microscopy, myonuclear lobulation with clumping of chromatin in the interior of the nucleus and condensation of chromatin in the nuclear envelope. The same infant had an extensive genetic workup for SMA, SMARD (SMA respiratory distress), sequencing of a congenital neuromuscular panel of 124 genes (including congenital myopathies, congenital myasthenia syndromes), as well as specific testing for Prader Willi, and myotonic dystrophy, which were all negative. Blood concentrations of zinc and alkaline phosphatase in the surviving patient were within normal limits: zinc concentration 85 mcg/dL (normal range 55–165 mcg/dL); alkaline phosphatase concentration 155 and 274 U/L at two different time points (normal range 48–406 U/L). Detailed clinical data for all affected individuals are given in Appendix S1.

### 3.2 Genetic studies

**FIGURE 2:**
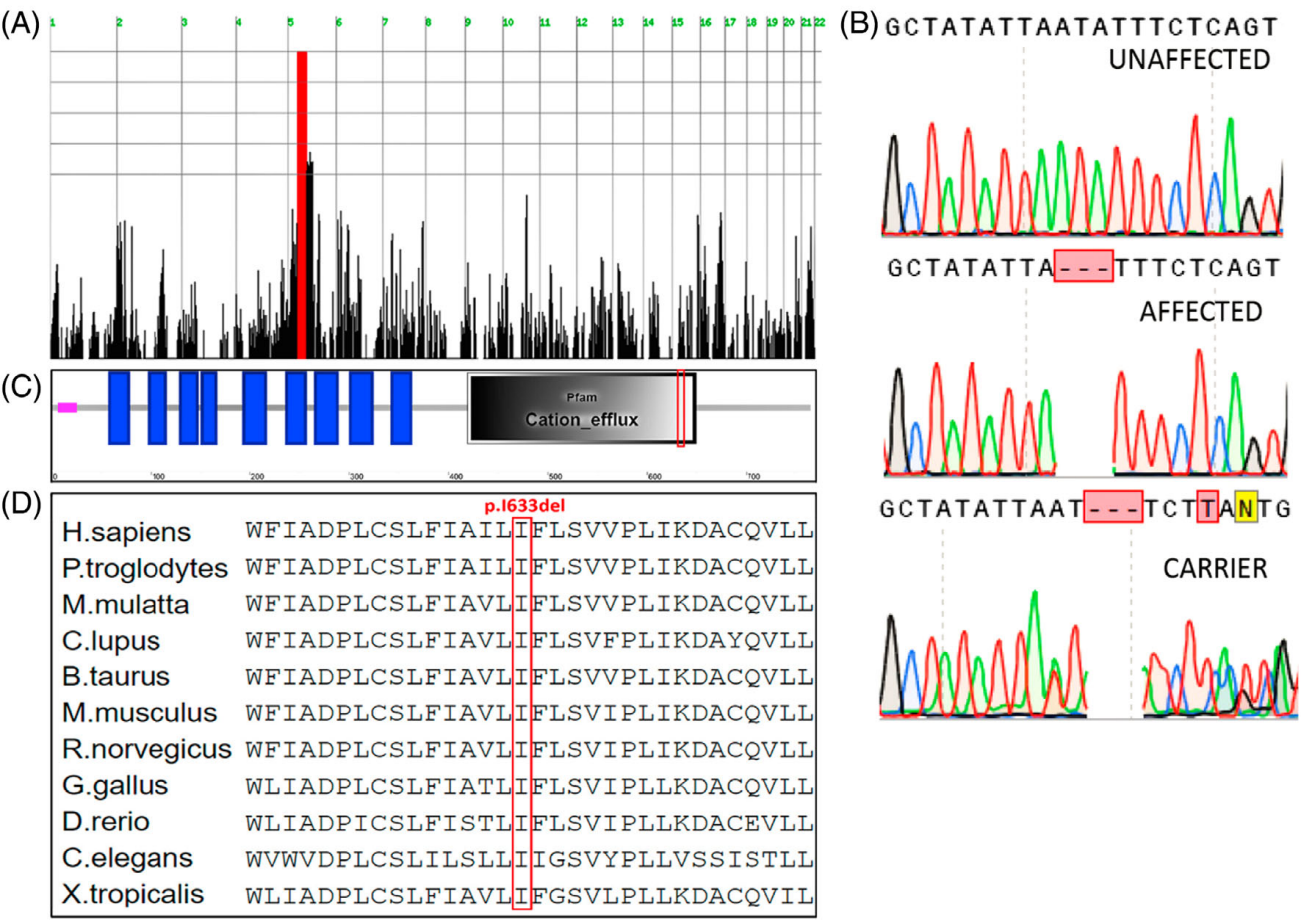
The *SLC30A5* variant: (A) homozygosity mapping: red marks a single chromosome 5 homozygous locus shared by all affected individuals (Homozygosity‐Mapper). (B) The c.1897_1899delATA *SLC30A5* variant (Sanger sequencing). (C) Protein effects of the variant: SMART diagram of SLC30A5 domain organization demonstrating that the deleted isoleucine (red rectangle) is within a conserved cation efflux domain, putatively disrupting its transmembrane helix. Blue rectangles represent transmembrane helix regions (per TMHMM v2.0 software); (D) evolutionary conservation of the mutated sequence: multiple protein sequence alignment of SLC30A5 and its putative homologs in different species (red rectangle highlights the deleted isoleucine).

Linkage analysis, testing individuals IV‐1,2, and V‐1,2,3,4,6,9,10 identified one distinct locus of homozygosity shared by and unique to all affected individuals: a chromosome 5p13.1‐q13.2 32.6 Mb disease‐associated locus (LOD score >3) between rs3099130 and rs3763153 (Figure 2A). WES data of IV‐3,4, V‐2,6,9 were filtered for normal variants as detailed in Section 2. Following exclusion of common variants (allele frequency >0.5% in the 1000 Genomes project, ExAC, NHLBI ESP exomes, CGI, or other datasets represented in Allele Frequency Community), only one homozygous variant (and no compound heterozygous variants) was identified within the chromosome 5 locus: a 3 bp in‐frame deletion: *SLC30A5* (NM_022902.5), c.1897_1899delATA (Figure 2B). This variant co‐segregated with the phenotype throughout all branches of the studied kindred, as was validated through Sanger sequencing (Figure 2B). It was predicted to be damaging by Mutation Taster, SIFT, and PolyPhen‐2 (CADD score 21.6) and had zero frequency in gnomAD and in our in‐house database of 1000 ethnically‐matched alleles. The *SLC30A5* variant causes deletion of a highly conservative isoleucine at position 633 of ZnT5, a member of the zinc‐transporters family, and is expected to affect its highly conserved cation efflux domain, putatively disrupting its transmembrane helix (Figure 2C).

### 3.3 Determination of the physiological effects of the SLC30A5 variant

**FIGURE 3:**
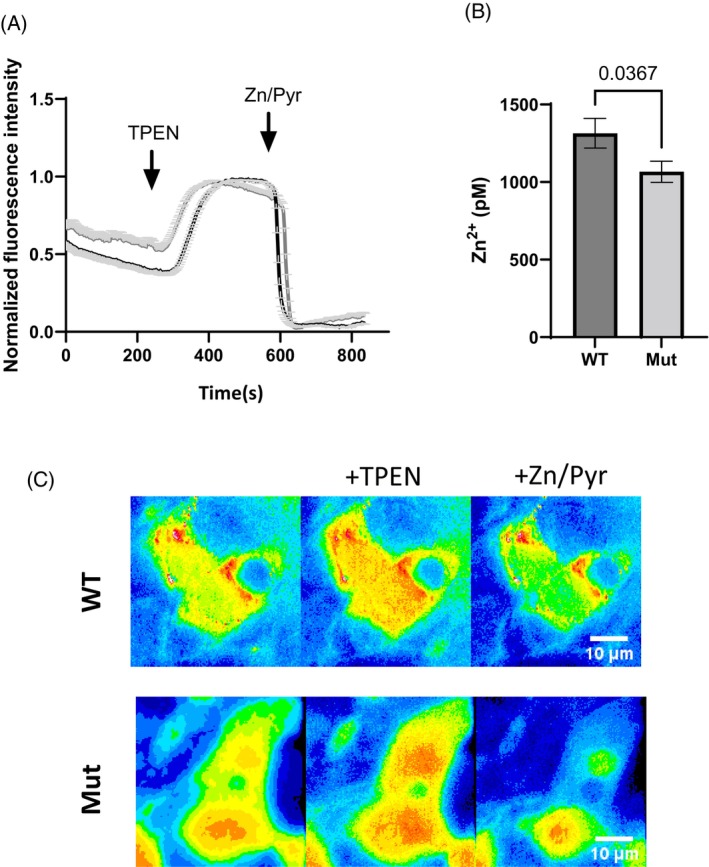
The c.1897_1899delATA *ZnT5* variant is associated with lower cytosolic zinc concentration: (A) normalized average traces obtained for one acquisition (*n* = 29 cells for HEK‐ZnT5‐WT and *n* = 22 cells for HEK‐ZnT5‐mut). (B) Free zinc concentration revealing significant decreases in cytosolic free zinc levels in cells expressing the mutant form compared to cells expressing wild‐type ZnT‐5 (two independent experiments, *n* = 97 cells for WT and *n* = 133 cells for mutant). Values are given as mean ± standard deviation. *p* = 0.0367. (C) Representative microscopy images for WT and mutant SLC30A5 overexpression in HEK293 cells. A further selection of images is given in Figure S1.

We went on to determine the functional consequences of the *SLC30A5* variant. As vesicular/ER zinc cannot be reliably measured with existing probes (due to competition with H^+^ in detecting zinc), we focused on measurements of cytosolic zinc. To that end we used the eCALWY family of genetically‐encoded FRET Zn^2+^ sensors, allowing measurements of free cytosolic zinc in transfected cells overexpressing either wild‐type or mutant ZnT5. Traces were obtained from two independent experiments and 5/6 acquisitions per condition. For each cell, cytosolic zinc concentration was calculated as follows: first, the steady‐state fluorescence intensity ratio (citrine to cerulean) was determined before obtaining the *R*_max_ under perifusion with KHB buffer containing the zinc chelator TPEN (50 mM; zinc‐free condition). Next, the *R*_min_ was obtained under perifusion with KHB buffer containing 5 mM pyrithione and 100 mM Zn^2+^ (zinc‐saturated condition), providing saturating intracellular Zn^2+^ concentrations (Figure 3A). Interestingly, there was a significant decrease in cytosolic free zinc levels in cells expressing the mutant form compared to cells expressing wild‐type ZnT5 (Figures 3B,C and S1).

## 4 DISCUSSION

Through studies of a consanguineous Bedouin kindred, we delineate an autosomal recessive disease of non‐progressive mostly axial hypotonia with severe head lag from birth and high arched palate, poor suck, weak neonatal primitive reflexes. Two of the five affected neonates required mechanical ventilation at birth, and progressive respiratory insufficiency, as well as FTT, were evident in the remaining three, requiring tube feeding through nasogastric tube or gastrostomy. None of the affected individuals survived beyond the age of 1 year. Notably, the hypotonia was not associated with seizures or cardiac or multi‐organ involvement. Extensive investigations, including brain imaging (MRI), metabolic and biochemical screening, karyotype, CMA, and genetic screening for known muscular dystrophies, myopathies, and myasthenic syndromes, failed to identify known diseases. Blood zinc and alkaline phosphatase were within normal limits. Homozygosity mapping combined with whole exome sequencing identified a likely disease‐causing 3 bp in‐frame deletion variant in *SLC30A5*, deleting a highly conserved isoleucine at position 633 within the highly‐conserved cation efflux domain of the encoded ZnT5, putatively disrupting its transmembrane helix. Functional studies through transfection experiments in HEK293 cells demonstrated that the *SLC30A5* variant results in significantly lower cytosolic Zn^2+^ concentrations.

Zinc ions, and the precise temporal control of their concentrations in various cellular compartments, are essential for the normal function of many human cell types. Chelatable zinc has been shown to be stored in the mitochondria, ER, and Golgi apparatus of living cells, as part of zinc homeostasis. While zinc concentrations in the mitochondria have been shown to be modulated by zinc transporters other than Znt5, Znt5 has been clearly shown to affect zinc stores in both the ER and Golgi.^22^, ^23^ The spatio‐temporal intracellular dynamics of zinc, crucial in cellular signaling, are mediated through coordinated actions of zinc transporters of the ZIP and ZnT families, regulating zinc redistribution between the cytosol and the ER lumen: the ubiquitously‐expressed ZnT5, ZnT6, and ZnT7 act in zinc supply into the ER, whereas ZIP7 controls zinc release from the ER into the cytosol.^4^ These factors play a role in alleviating ER stress and apoptosis: zinc‐limited mutants of MSC2, the yeast ortholog of mammalian Znt5, exhibit UPR activation and defective ER‐associated protein degradation; moreover, in chicken DT40 lymphoma cells, double deletions of *ZnT5* and *ZnT7* induce UPR due to defective zinc sequestration into the ER lumen, suggesting that both excess and deficiency of zinc in the ER could induce ER stress.^1^

Znt5 also plays a role in controlling zinc concentrations in the Goli apparatus. While most ZnTs and ZIPs function as homodimers, ZnT5 and ZnT6 heterodimerize: ZnT5 acts as an operative zinc transporter and carries the sorting signal directing the ZnT5–ZnT6 heterodimer to the Golgi apparatus, while ZnT6 is a putative auxiliary protomer in the heterodimer.^24^ Three membrane transporter complexes, ZnT4, ZnT5/ZnT6, and ZnT7, import Zn^2+^ into the Golgi lumen in exchange with protons. Specifically, ZnT5/Znt6 heterodimers regulate labile Zn^2+^ concentration at the medial Golgi, consistent with their localization. In fact, ZnT‐mediated Zn^2+^ fluxes tune the localization, trafficking, and client‐retrieval activity of ERp44 (a chaperone operating in the early secretory pathway), controlling Zn^2+^ homeostasis and ERp44‐mediated proteostasis along the early secretory pathway.^25^ ZnT5–ZnT6 heterodimers and ZnT7 homodimers also constitute zinc‐supplying routes to nascent ectoenzymes in the early secretory pathway, activating several specific zinc ectoenzymes, including alkaline phosphatases (ALPs), Ecto‐5′‐nucleotidase and Autotaxin (ATX, termed also ectonucleotide pyrophosphatase/phosphodiesterase 2, ENPP2). Zinc has been shown to act as a cofactor for ALP activation.^26^ Moreover, some zinc ectoenzymes cannot stably exist in an apo‐form in cells lacking both ZnT5–6 and ZnT7 dimers, indicating that they are also closely associated with protein quality control of ectoenzymes. For instance, degradation of ALPs through ubiquitin‐proteasomal and lysosomal degradation pathways in ZnT5, ZnT6, and ZnT7 triple deficiency cells is irreversible even if excess zinc is added to the cell culture. Zn*T5* transcription is induced by ER stress through the XBP‐1 transcription factor in HeLa cells, mouse ES cells, and mouse MIN6 pancreatic β cells, consistent with the notion that ZnT5–6 and ZnT7 indeed contribute to protein quality control in the early secretory pathway. Post‐translational modifications may also contribute to ZnT‐dependent stability of ALPs^1^: ZnT5–6 and ZnT7 also supply zinc to zinc proteins other than zinc ectoenzymes, such as the ER chaperone ERp44, whose C‐terminal conformation is responsive to zinc binding, driving ER–Golgi retrograde transport of ERp44 accompanied by the capture and retrieval of client proteins such as Ero1α and ERAP1.^1^

The neurologic phenotype seen in the affected individuals is in line with the role of ZnT5 in neuronal development and function. Zinc transport mechanisms have been shown to act extensively in embryonic brain development.^2^ Moreover, intracellular zinc, highly concentrated at synaptic boutons, has been shown to play many roles in the brain, from affecting neurotransmission and sensory processing, to activating both pro‐survival and pro‐death neuronal signaling pathways. Zinc levels in various intracellular compartments, determining those activities, are tightly regulated by metal binding proteins and a large array of zinc transporters.^27^ Specifically, unlike ZnT1 or ZnT2–4 which localize to the plasma membrane or intracellular vesicles respectively, ZnT5 (and 7) localize to the Golgi apparatus, likely active in maintaining Golgi zinc when cytosolic levels are depleted.^27^

The affected individuals had also dramatic FTT, possibly due to a role of ZnT5 in normal function of the gastrointestinal system, as evidenced by high levels of ZnT5 expression in parietal cells of the stomach and in the absorptive epithelium of the duodenum and jejunum.^28^ The FTT seen in the human patients is in line with the phenotype of *ZnT5* null‐mutant mice^29^ that showed poor growth and a decrease in bone density due to impairment of osteoblast maturation to osteocyte.^29^ However, while ZnT5‐deficient mice showed growth defects as well as osteopenia, muscle weakness, and male‐specific cardiac death, they did not suffer from global Zn deficiency, showing that ZnT5 is not essential for Zn absorption.

In ZnT5 null‐mutant mice, 60% of male homozygotes die of cardiac brady‐arrhythmias.^29^ Similarly, previously‐reported human individuals homozygous for *SLC30A5* biallelic truncation variants (p.(Ile278Phefs*33) and p.(His661Tyrfs*10), NM_022902.4), putatively causing loss of function, presented with cardiomyopathy and perinatal death. These four patients reported to date were born prematurely (gestational weeks 27–31) and died between 31 weeks of gestation to the 5th day of life. All had hydrops fetalis/cystic hygroma noted between week 22 and 31 of gestation, most likely secondary to cardiomyopathy (delineated as non‐compaction cardiomyopathy in one patient), with arrhythmias, mostly bradycardia, in two. There were no evident neurological anomalies, aside from brain imaging showing findings likely secondary to hygroma/hemorrhage in one of the individuals.^30^ Notably, causation of the disease phenotype by the *SLC30A5* variants was not proven statistically or through functional studies.^30^

In contrast to the four previously reported cases with *SLC30A5* variants, our patients did not present with hydrops fetalis/cystic hygroma or any evident cardiac phenotype, yet had severe neurological manifestations. Moreover, all affected individuals in our cohort survived several months—up to nearly a year, though major intervention was necessary. Of our five patients, four were male and died and the only one female is yet alive (though only 9 months old at present). While these data are possibly in line with the mouse studies reporting male‐specific death in *Znt5* null‐mutants, our human data are far from sufficient to suggest such male predilection. Moreover, in the previous report of perinatal death associated with homozygous *SLC30A5* variants, two of the four affected individuals were females.^30^

ZnT5 is ubiquitously expressed, and acts in retention of Zn^2+^ ions in differential subcellular localization (either in the Golgi apparatus or throughout the cell, including at the plasma membrane), which is isoform dependent.^31^, ^32^ Different splice variants of *SLC30A5* encode for proteins with different subcellular localizations, that are regulated by zinc through transcription and mRNA stability.^31^, ^32^ The differential subcellular localization of the splice variants is dictated by the different C‐terminal regions.^27^ Interestingly, the human ZnT5 variant B (ZnT5B (hZTL1)) is localized to the plasma membrane, where it functions as a Zn exporter, similarly to other *SLC30A* family members, but also as a Zn uptake regulator.^33^ Through measurements of free cytosolic Zn^2+^ in HEK293 cells overexpressing SLC30A5, we showed a significant decrease in cytosolic free Zn^2+^ levels in cells expressing the in‐frame three nucleotide deletion mutant form (found in our patients) compared to cells expressing wild‐type ZnT5. ZnT proteins are known to reduce cytosolic zinc concentration, either through efflux across the plasma membrane or through sequestration in intracellular compartments. However, specific properties of different Zn^2+^ transporters still need to be studied. In fact, our findings align with some observations^33^ that ZnT5 functions bi‐directionally and can regulate the cytosolic concentration of zinc by allowing Zn^2+^ into the cytosol, wherein the mutant is less active. These findings are also in line with our previous study^12^ showing that an autosomal recessive cerebro‐renal syndrome with neurological regression evident from early age, progressing into severe intellectual disability/developmental delay, is caused by an in‐frame deletion of 3 bp in *SLC30A9* (also known as *ZnT9*). Interestingly, similar to our findings with *SLC30A5*, cytosolic Zn^2+^ measurements in HEK293 cells overexpressing wild‐type and mutant *SLC30A9* showed lower zinc concentration within mutant rather than wild‐type *SLC30A9* cells. Moreover, the *SLC30A9* variant is expected to affect its highly conserved cation efflux domain, putatively disrupting its transmembrane helix structure, which strikingly resembles the findings of our current study on *SLC30A5*.

While a loss‐of‐function effect of the ZnT5 variant is plausible, there are other possibilities as to the precise mechanism of the disease process: for instance, the missense variant might confer gain‐of‐function of ZnT5. Alternatively, the *ZnT5* missense variant might impact the interaction of ZnT5 with ZnT6: normally, ZnT5 and ZnT6 need to be co‐expressed to transport zinc to activate tissue‐nonspecific alkaline phosphatase (TNAP).^34^ Activation of TNAP occurs at the ER due to glycosylphosphatidylinositol (GPI) modification at the C‐terminus producing GPI‐anchored protein, where ZnT5–ZnT6 hetero‐dimers play a pivotal role as key Zn^2+^ providers. GPI‐anchored proteins play crucial roles in various enzyme activities, cell signaling and adhesion, and immune responses. The early secretory pathway ZNTs (ZNT5–ZNT6 heterodimers and ZNT7 homodimers), which supply zinc to the lumen of the early secretory pathway compartments, are essential for GPI‐anchored protein expression on the cell surface.^35^ To date, more than 150 GPI‐anchored proteins are characterized in humans and function as enzymes, receptors, adhesion proteins, and complement regulatory proteins on the cell surface.^35^ Mutations in multiple phosphatidylinositol glycan anchor biosynthesis (PIG) genes are associated with defects in GPI‐anchored proteins (inherited GPI deficiency), resulting in prominent human diseases including intellectual disability, hypotonia, facial dysmorphism, seizures, and dystonia.^36^ Thus, it is plausible that the variant in our patients affects interaction of ZnT5 with ZnT6, altering activation of TNAP. Such an effect would differ from that of null mutations in *ZnT5*, as expected in the previously reported *ZnT5* frameshift truncation mutations (whose functional effects remain to be elucidated) and might explain the different phenotypes.

Finally, it should be noted that *ZnT5* polymorphisms have also been statistically associated with various pathological phenotypes in humans, including an increase in inflammatory markers, obesity, type 2 diabetes mellitus, colorectal cancer, and Alzheimer's disease.^35^ While none of these phenotypes were found in our patients or in the previously reported human *ZnT5* homozygous truncation mutants, their early death has possibly precluded the appearance of such phenotypes.

Altogether, we demonstrate that a bi‐allelic in‐frame deletion missense variant in the zinc carrier SLC305A (ZnT5) results in lower cytosolic Zn^2+^ concentrations, causing a severe syndrome of neonatal non‐progressive mostly axial yet also limb hypotonia with failure to thrive, and respiratory failure culminating in early death within the first year of life.

## AUTHOR CONTRIBUTIONS

Planning, conception, and design of the study: VD, PC, GAR, KM, OSB. Conduct, acquisition, and analysis of data: all authors. Reporting—writing the manuscript: VD, OSB with contributions and corrections of all authors.

## FUNDING INFORMATION

The study was funded by the Israel Science Foundation (grant no. 2034/18) awarded to OSB, the Morris Kahn Family Foundation, and the Israeli Ministry of Science and Technology National Center for Rare Diseases. GAR was supported by a Wellcome Trust Investigator Award (212625/Z/18/Z), UK MRC Programme grant (MR/R022259/1), Diabetes UK Project grant (BDA16/0005485), CRCHUM start‐up funds, an Innovation Canada John R. Evans Leader Award (CFI 42649), and NIH‐NIDDK (R01DK135268) project grant.

## CONFLICT OF INTEREST STATEMENT

Vadim Dolgin, Pauline Chabosseau, Jacob Bistritzer, Iris Noyman, Orna Staretz‐Chacham, Ohad Wormser, Noam Hadar, Marina Eskin‐Schwartz, Bibi Kanengisser‐Pines, Ginat Narkis, Ramy Abramsky, Eilon Shany, Guy A. Rutter, Kyla Marks, and Ohad S. Birk declare that they have no conflict of interest. Guy A. Rutter has received grant funding from, and is a consultant for, Sun Pharmaceuticals Industries Ltd. This company was not involved in any way in the design or execution of the present study.

## INFORMED CONSENT

All procedures followed were in accordance with the ethical standards of the responsible committee on human experimentation (institutional and national) and with the Helsinki Declaration of 1975, as revised in 2000. Informed consent was obtained from all patients for being included in the study. Proof that informed consent was obtained is available upon request.

## Supporting information


**FIGURE S1.** The c.1897_1899delATA *ZnT5* variant is associated with lower cytosolic zinc concentration: Representative microscopy images for WT and mutant SLC30A5 overexpression in HEK293 cells. A further selection of images.

Appendix S1.

## Data Availability

All data are available from the corresponding author upon request.
