# Supplementary material for: Severe neonatal hypotonia due to SLC30A5 variant affecting function of ZnT5 zinc transporter
Source: JIMD Rep. 2025 Jan 9;66(1):e12465. doi: 10.1002/jmd2.12465 (PMC11712426; doi:10.1002/jmd2.12465)

**Supplementary Figure 1: The c.1897_1899delATA *ZnT5* variant is associated with lower cytosolic zinc concentration:** Representative microscopy images for WT and mutant SLC30A5 overexpression in HEK293 cells. A further selection of images.


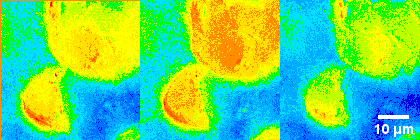

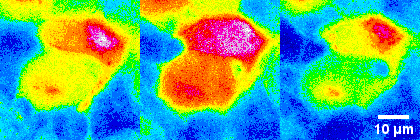

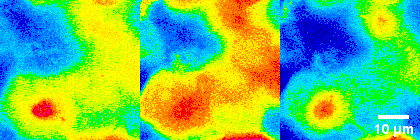


**WT**

+TPEN

+Zn/Pyr

**WT**

**WT**


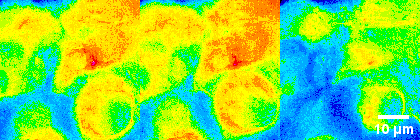

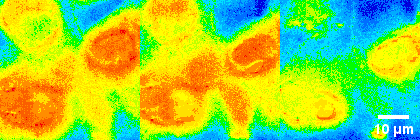


**Mut**

**Mut**

**Mut**


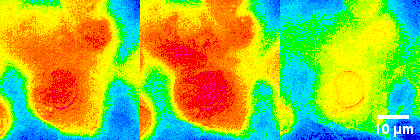

Supplement: Supplementary file 1 — FIGURE S1. The c.1897_1899delATA ZnT5 variant is associated with lower cytosolic zinc concentration: Representative microscopy images for WT and mutant SLC30A5 overexpression in HEK293 cells. A further selection of images. [file JMD2-66-e12465-s002.docx]
