# Supplementary material for: Severe neonatal hypotonia due to SLC30A5 variant affecting function of ZnT5 zinc transporter
Source: JIMD Rep. 2025 Jan 9;66(1):e12465. doi: 10.1002/jmd2.12465 (PMC11712426; doi:10.1002/jmd2.12465)
Supplement: Supplementary file 2 — Appendix S1. [file JMD2-66-e12465-s001.docx]

Supplementary material:

Clinical details of the affected individuals:

Individual V-2 was born late preterm (35^+6^ weeks) following PROM and was discharged from the postnatal ward at one week. On physical exam he had severe head lag and axial hypotonia with normal neonatal primitive and deep tendon reflexes without tongue fasciculations. Urinary organic acids, as well as blood amino acids, cholesterol, transferrin iso-electric focusing, very long chain fatty acids (VLCFA), lactate/pyruvate, carnitine, blood gases, creatinine phosphokinase (CPK) and glucose, were within normal limits. Karyotype was normal. Chest x-ray demonstrated small lung volumes. He was admitted to the Pediatric Intensive Care Unit via the emergency room at age two months and succumbed shortly afterward.

V-3, brother of V-2, was born at term (birth weight 2.8kg) by caesarean section (CS) following fetal distress. He was admitted to the neonatal intensive care unit (NICU) at 24 hours of age with increased oxygen requirements and respiratory acidosis, and responded to treatment with non-invasive ventilation. On physical examination he had mild head lag and axial hypotonia without dysmorphic features. Chest X-ray revealed restricted lung expansion (less than five ribs) and a globular heart occupying most of the lung fields. Head ultrasound demonstrated mild ventriculomegaly and partial/thin corpus callosum; brain MRI was not performed. Urinary and blood tests, similar to those of V-2, were normal, as were echocardiography and thyroid function tests. Karyotype and molecular testing for SMA and Prader Willi were normal. In the following six months, he was repeatedly admitted to the hospital with FTT (weight <<3^rd^ percentile age 2-7m), vomiting, respiratory distress and fever. Nasogastric feeds were commenced but failed to improve the FTT. At seven months, he contracted Covid 19, required admission to the pediatric intensive care unit, and succumbed to respiratory failure at 8.5 months.

V-6 was born at 42^+6^ weeks (birth weight 3.965 Kg) following an uneventful pregnancy – with no abnormal fetal movements or polyhydramion. Hypotonia and severe respiratory distress were evident at birth, as were high-arched palate and large anterior fontanelle. Mechanical ventilation was required as of birth. Repeated neurological exams (last at age 9 months) suggested combined upper and lower motor neuron involvement, with brisk tendon reflexes and clonus, choreiform movements, as well as severe hypotonia. DQ at 9 months was below 50. Chest X ray (Fig. 1B) demonstrated restricted lung expansion (less than six ribs). An extensive array of blood tests (including extensive biochemical tests, CPK, cortisol, growth hormone and prolactin), bronchoscopy, ECHO-cardiography, cardiac ECG and holter, as well as brain MRI, were all normal. EEG at 9 months demonstrated low voltage non-variable background with no epileptiform activity. Sequencing of a panel of 124 genes associated with neuromuscular diseases, as well as segregation analysis of few variants of unknown significance, failed to identify a likely disease-associated variant. Specific genetic testing for SMA and myotonic dystrophy, as well as CMA – were normal.

V-9 was born at term (37 weeks, 3.050 Kg) following an uneventful pregnancy. Birth was through emergency CS following fetal distress. On physical exam at birth he was hypotonic with significant head lag. Extensive blood and urine tests (including blood magnesium, CPK, TSH, testosterone) were within normal limits. Genetic work-up was negative for Prader Willi, and CMA was with no significant abnormal findings. Nasogastric feeds were commenced at 17 days. He was admitted repeatedly with poor weight gain, vomiting, and respiratory distress due to aspiration pneumonia, and succumbed at age 1yr, one month following massive aspiration, cardiac arrest, and significant anoxic brain injury.

V-10 was born at term (41 weeks; 3.560 Kg) with pulmonary hypertension (requiring NO treatment) and no respiratory effort with resolution of pulmonary hypertension after two days. Repeated extubation attempts failed, and long-term ventilation through tracheostomy was instituted. Hypotonia (mostly axial) and large anterior fontanelle were evident. Deep tendon reflexes were normal or brisk and there were no tongue fasciculations. At present age 9 months, she is fed through a nasogastric tube and is chronically ventilated, FTT is evident as are severe neurodevelopmental delay and hypotonia.
